# Genetic predictors of response to specific drugs in primary myelofibrosis

**DOI:** 10.1038/s41408-018-0158-4

**Published:** 2018-11-19

**Authors:** Domenico Penna, Natasha Szuber, Terra L. Lasho, Christy M. Finke, Rangit R. Vallapureddy, Curtis A. Hanson, Rhett P. Ketterling, Animesh Pardanani, Naseema Gangat, Ayalew Tefferi

**Affiliations:** 1 Divisions of Hematology, Departments of Internal Medicine and Laboratory Medicine, Mayo Clinic, Rochester, MN USA; 2 Divisions of Hematopathology, Departments of Internal Medicine and Laboratory Medicine, Mayo Clinic, Rochester, MN USA; 3 Divisions of Laboratory Genetics and Genomics, Departments of Internal Medicine and Laboratory Medicine, Mayo Clinic, Rochester, MN USA

Primary myelofibrosis (PMF) constitutes clonal expansion of myeloid cells and is characterized by “driver” (i.e., *JAK2*, *CALR*, and *MPL*) and other mutations or DNA variants, such as *ASXL1*, *TET2*, *SRSF2*, *IDH1*, *IDH2*, and *U2AF1*^1–3^. Patients with PMF have an estimated median survival of 6 years and causes of death include leukemic transformation; in addition, quality of life is markedly impaired in PMF as result of frequent red blood cell transfusion requirement, markedly enlarged spleen and severe constitutional symptoms^4,5^. At present allogeneic stem cell transplant (ASCT) is the only treatment modality with potential to either cure the disease or prolong survival of patients with PMF, while current drug therapy offers palliative value only (e.g., reduction in spleen size, reduction of constitutional symptoms, and improvement in anemia)^6,7^. In this regard, hydroxyurea (HU), JAK2 inhibitors, and interferon (IFN)-α are often used for symptomatic splenomegaly: JAK2 inhibitors for constitutional symptoms and immunomodulatory drugs (IMiDs), erythropoiesis-stimulating agents (ESAs), and androgens for anemia^8^.

The current study was approved by the Mayo Clinic Institutional Review Board (IRB) and represents a retrospective evaluation of specific drug response, in terms of anemia or splenomegaly, in patients with PMF receiving these agents as first-line treatment. Diagnoses and treatment approaches were in accordance with what was considered standard of care at the time of initial diagnosis or first referral^9^. Study patients were recruited from the Mayo Clinic, Rochester, MN, USA. Diagnoses were according to the 2016 World Health Organization criteria^10^. To ascertain the role of genetic biomarkers, study inclusion criteria included availability of karyotype and next-generation sequencing (NGS)-derived mutation information.

Variables evaluated included those that are currently listed in MIPSS70 (mutation-enhanced international prognostic scoring system for transplant-age patients), MIPSS70 + *version 2.0* (karyotype-enhanced MIPSS70), and GIPSS (genetically-inspired prognostic scoring system)^11–13^. For the purposes of the current study, conventional response criteria were modified to reflect clinical benefit assessment without strict adherence to criteria designed for clinical trials. Accordingly, spleen response was evaluated only in patients with palpable splenomegaly and was defined as a minimum 50% reduction in palpable spleen size, regardless of response duration. Anemia response was evaluated only in patients with hemoglobin level < 10 g/dl and was defined as achieving transfusion-independence lasting for at least 1 month or an increase in hemoglobin of 2 g/dl, regardless of response duration^14^. Statistical analyses considered clinical and laboratory parameters obtained at time of diagnosis or first referral, which coincided, in all instances, with time of sample collection for mutation analysis. Conventional methods were used for statistical analysis (JMP® Pro 13.0.0 software; SAS Institute, Cary, NC).

A total of 432 cytogenetically- and molecularly-annotated patients with PMF were accessed (Supplementary Table 1) in order to identify 333 patients who received first-line treatment with HU (*n* = 97), JAK2 inhibitors (*n* = 41), IFN-α (*n* = 22), IMiDs (*n* = 58), androgens (*n* = 19), ESAs (*n* = 54), and various other drugs (*n* = 42), and were evaluable for response. Presenting features of the 333 study patients (median age 64 years; 68% males) included palpable splenomegaly (77%), moderate to severe anemia (58%), constitutional symptoms (31%), platelet count < 100 × 10^9^/l (20%), leukocytosis > 25 × 10^9^/l (14%), and ≥ 2% circulating blasts (30%). Driver mutational status was *JAK2* 59%, *CALR* type 1/like 20%, *CALR* type 2/like 4%, *MPL* 7%, and triple-negative 10%. Karyotype included very high risk (VHR) 6%, unfavorable 18%, and favorable 76%, according to the recently revised system^15^. Sixty percent of the patients harbored high molecular risk (HMR) mutations, including *ASXL1* (45%), *SRSF2* (18%), *U2AF1*Q157 (10%), *EZH2* (4%), *IDH2* (4%), and *IDH1* (2%). MIPSS70 + *version 2.0* risk distribution was as follows: very high 17%, high 46%, intermediate 21%, low 14%, and very low 2%.

Overall, 249 patients were evaluable for spleen response, including 218 that were treated with the specific drugs analyzed in the current study (Table 1). Anemia response was evaluated in 222 patients (118 were red cell transfusion-dependent), including 194 (105 transfusion-dependent) that were treated with the specific drugs analyzed in the current study (Table 2).

**Table 1:** Predictors of spleen response to specific drugs in patients with primary myelofibrosis receiving these agents as first-line treatment

Baseline characteristics	HU (*n* = 73)	Response (*n*; %)	*P* value	JAK2 inhibitors (*n* = 40)	Response (*n*; %)	*P* value	IFN-α (*n* = 17)	Response (*n*; %)	*P* value	IMiDs (*n* = 41)	Response (*n*; %)	*P* value	Androgens (*n* = 11)	Response (*n*; %)	*P* value	ESAs (*n* = 36)	Response (*n*; %)	*P* value
*Age*																		
>70 years; *n (%)*≤	17 (23)	4 (24)	0.7	6 (15)	3 (50)	0.6	*1 (6)	1 (100)	0.1	12 (29)	3 (25)	0.3	4 (36)	1 (25)	0.3	15 (42)	0	-
70 years; *n (%)*	56 (77)	16 (29)		34 (85)	23 (68)		16 (94)	2 (12)		29 (71)	4 (14)		7 (64)	0		21 (58)	0	
*Sex*																		
Males; *n (%)*	51 (70)	16 (31)	0.3	24 (60)	12 (50)	**0.01**	8 (47)	2 (25)	0.5	30 (73)	4 (13)	0.3	8 (73)	1 (12)	1	22 (61)	0	-
Females; *n (%)*	22 (30)	4 (18)		16 (40)	14 (87)		9 (53)	1 (11)		11 (27)	3 (27)		*3 (27)	0		14 (39)	0	
*Moderate/severe anemia (sex-adjusted)*																		
Presence; *n (%)*	34 (47)	6 (18)	0.1	14 (35)	8 (57)	0.5	6 (35)	1 (17)	1	33 (81)	7 (21)	0.3	9 (82)	1 (11)	1	26 (74)	0	-
Absence; *n (%)*	39 (53)	14 (36)		26 (65)	18 (69)		11 (65)	2 (18)		8 (19)	0		*2 (18)	0		9 (26)	0	
*Transfusion dependency*																		
Presence; *n (%)*	16 (22)	4 (25)	1	8 (20)	4 (50)	0.4	4 (23)	1 (25)	1	24 (59)	4 (17)	1	4 (36)	0	1	18 (50)	0	-
Absence; *n (%)*	57 (78)	16 (28)		32 (80)	22 (69)		13 (76)	2 (15)		17 (41)	3 (18)		7 (64)	1 (14)		18 (50)	0	
*Platelets*																		
<100 × 10^9^/L; *n (%)*	11 (15)	1 (9)	0.2	5 (12)	2 (40)	0.3	5 (29)	1 (20)	1	12 (29)	1 (8)	0.6	6 (55)	1 (17)	1	7 (19)	0	-
≥100 × 10^9^/L; *n (%)*	62 (85)	19 (31)		35 (88)	24 (35)		12 (71)	2 (17)		29 (71)	6 (21)		5 (45)	0		29 (81)	0	
*Leukocytes*																		
>25 × 10^9^/L; *n (%)*	15 (21)	2 (13)	0.1	6 (15)	2 (33)	0.1	3 (18)	0	1	5 (12)	0	0.5	*2 (18)	0	1	6 (17)	0	-
≤25 × 10^9^/L; *n (%)*	58 (79)	18 (31)		34 (85)	24 (71)		14 (82)	3 (21)		36 (88)	7 (19)		9 (82)	1 (11)		29 (83)	0	
*Circulating blasts*																		
≥2%; *n (%)*	21 (29)	4 (19)	0.5	16 (41)	9 (56)	0.5	6 (35)	0	0.5	14 (34)	1 (7)	0.3	3 (27)	0	1	12 (33)	0	-
<2%; *n (%)*	51 (71)	15 (29)		23 (59)	16 (70)		11 (65)	3 (27)		27 (66)	6 (22)		8 (73)	1 (12)		24 (67)	0	
*Constitutional symptoms*																		
Presence; *n (%)*	27 (37)	6 (22)	0.5	13 (32)	8 (62)	1	4 (24)	1 (25)	1	15 (37)	2 (13)	1	3 (27)	0	1	12 (33)	0	-
Absence; *n (%)*	46 (63)	14 (31)		27 (68)	18 (67)		13 (76)	2 (15)		26 (63)	5 (19)		8 (73)	1 (12)		24 (67)	0	
*Palpable splenomegaly*																		
Presence; *n (%)*	63 (86)	16 (25)	0.4	33 (82)	23 (70)	0.2	15 (88)	3 (20)	1	39 (95)	7 (18)	1	9 (82)	0	0.1	31 (86)	0	-
Absence; *n (%)*	10 (14)	4 (40)		7 (18)	3 (43)		2 (12)	0		*2 (5)	0		*2 (18)	1 (50)		5 (14)	0	
*Favorable karyotype*																		
Presence; *n (%)*	53 (73)	17 (32)	0.2	32 (80)	20 (62)	0.6	14 (82)	3 (21)	1	29 (71)	6 (21)	0.6	9 (82)	1 (11)	1	27 (75)	0	-
Absence; *n (%)*	20 (27)	3 (15)		8 (20)	6 (75)		3 (18)	0		12 (29)	1 (8)		*2 (18)	0		9 (25)	0	
*Unfavorable karyotype*																		
Presence; *n (%)*	15 (21)	3 (20)	0.7	8 (20)	6 (75)	0.6	*0	-	-	7 (17)	0	0.3	*2 (18)	0	1	6 (17)	0	-
Absence; *n (%)*	58 (79)	17 (29)		32 (80)	20 (62)		17 (100)	3 (18)		34 (83)	7 (21)		9 (82)	1 (11)		30 (83)	0	
*VHR karyotype*																		
Presence; *n (%)*	5 (7)	0	0.3	*0	-	-	3 (18)	0	1	5 (12)	1 (20)	1	*0	-	-	3 (8)	0	-
Absence; *n (%)*	68 (93)	20 (29)		40 (100)	26 (65)		14 (82)	3 (21)		36 (88)	6 (17)		11 (100)	1 (9)		33 (92)	0	
*JAK2 mutation*																		
Presence; *n (%)*	53 (73)	13 (25)	0.3	25 (62)	16 (64)	1	8 (47)	2 (25)	0.5	22 (54)	3 (14)	0.6	5 (45)	1 (20)	0.4	19 (53)	0	-
Absence; *n (%)*	20 (27)	7 (35)		15 (38)	10 (67)		9 (53)	1 (11)		19 (46)	4 (21)		6 (55)	0		17 (47)	0	
*CALR type-1/like mutation*																		
Presence; *n (%)*	12 (16)	4 (33)	0.7	10 (25)	9 (90)	0.06	6 (35)	1 (17)	1	12 (29)	3 (25)	0.3	*2 (18)	0	1	6 (17)	0	-
Absence; *n (%)*	61 (84)	16 (26)		30 (75)	17 (57)		11 (65)	2 (18)		29 (71)	4 (14)		9 (82)	1 (11)		30 (83)	0	
*CALR type-2/like mutation*																		
Presence; *n (%)*	3 (4)	2 (67)	0.1	*0	-	-	*1 (6)	0	1	*1 (2)	0	1	*1 (9)	0	1	*2 (6)	0	-
Absence; *n (%)*	70 (96)	18 (26)		40 (100)	26 (65)		16 (94)	3 (19)		40 (98)	7 (17)		10 (91)	1 (10)		34 (94)	0	
*MPL mutation*																		
Presence; *n (%)*	2 (3)	1 (50)	0.4	*1 (3)	1 (100)	1	*1 (6)	0	1	3 (7)	1 (33)	0.4	*1 (9)	0	1	4 (11)	0	-
Absence; *n (%)*	71 (97)	19 (27)		39 (97)	25 (64)		16 (94)	3 (19)		38 (93)	6 (16)		10 (91)	1 (10)		32 (89)	0	
*Triple-negative*																		
Presence; *n (%)*	3 (4)	0	0.5	4 (10)	0	**0.01**	*1 (6)	0	1	3 (7)	0	1	*2 (18)	0	1	5 (14)	0	-
Absence; *n (%)*	70 (96)	20 (29)		36 (90)	26 (72)		16 (94)	3 (19)		38 (93)	7 (18)		9 (82)	1 (11)		31 (86)	0	
*ASXL1 mutation*																		
Presence; *n (%)*	33 (45)	13 (40)	0.06	20 (50)	13 (65)	1	7 (41)	0	0.2	20 (49)	3 (15)	1	3 (27)	0	1	19 (53)	0	-
Absence; *n (%)*	40 (55)	7 (17)		20 (50)	13 (65)		10 (59)	3 (30)		21 (51)	4 (19)		8 (73)	1 (12)		17 (47)	0	
*SRSF2 mutation*																		
Presence; *n (%)*	15 (21)	7 (47)	0.1	13 (32)	7 (54)	0.4	3 (18)	1 (33)	0.4	10 (24)	0	0.1	*0	-	-	4 (11)	0	-
Absence; *n (%)*	58 (79)	13 (22)		27 (68)	19 (70)		19 (82)	2 (14)		31 (76)	7 (23)		11 (100)	1 (9)		32 (89)	0	
*EZH2 mutation*																		
Presence; *n (%)*	3 (4)	1 (33)	1	*0	-	-	*1 (6)	0	1	*2 (5)	0	1	*1 (9)	0	1	3 (8)	0	-
Absence; *n (%)*	70 (96)	19 (27)		40 (100)	26 (65)		16 (94)	3 (19)		39 (95)	7 (18)		10 (91)	1 (10)		33 (92)	0	
*IDH1 mutation*																		
Presence; *n (%)*	3 (3)	2 (67)	0.1	*1 (2)	1 (100)	1	*0	-	-	*0	-	-	*0	-	-	*2 (6)	0	-
Absence; *n (%)*	70 (97)	18 (26)		39 (98)	25 (64)		17 (100)	3 (18)		41 (100)	7 (17)		11 (100)	1 (9)		34 (94)	0	
*IDH2 mutation*																		
Presence; *n (%)*	4 (5)	0	0.5	*1 (2)	0	0.3	*0	-	-	3 (7)	0	1	*0	-	-	3 (8)	0	-
Absence; *n (%)*	69 (95)	20 (29)		39 (98)	26 (67)		17 (100)	3 (18)		38 (93)	7 (18)		11 (100)	1 (9)		33 (92)	0	
*U2AF1* Q157																		
Presence; *n (%)*	4 (5)	0	0.5	*1 (2)	0	0.3	*1 (6)	0	1	6 (15)	0	0.5	*1 (9)	1 (100)	0.09	4 (11)	0	-
Absence; *n (%)*	69 (95)	20 (29)		39 (98)	26 (67)		16 (94)	3 (19)		35 (85)	7 (20)		10 (91)	0		32 (89)	0	
*High molecular risk mutations*																		
Presence; *n (%)*	46 (63)	17 (37)	**0.02**	26 (65)	15 (58)	0.2	10 (59)	1 (10)	0.5	30 (73)	3 (10)	0.06	5 (45)	1 (20)	0.4	23 (64)	0	-
Absence; *n (%)*	27 (37)	3 (11)		14 (35)	11 (79)		7 (41)	2 (29)		11 (27)	4 (36)		6 (55)	0		13 (36)	0	

The values in bold indicate a significant *P* value ( < 0.05)

The values preceded by asterisk indicate an insufficient number of patients in the sample ( ≤ 2)

*JAK2* Janus kinase 2, *CALR* Calreticulin, *MPL* MPL proto-oncogene, *ASXL1* additional sex combs like 1, *SRSF2* Serine/arginine-rich splicing factor 2, *U2AF1* U2small nuclear RNA auxiliary factor 1, *EZH2* enhancer of zeste homolog 2, *IDH1/2* isocitrate dehydrogenase ½, *HU* Hydroxyurea, *IFN-α* Interferon α, *IMiDs* Immuno-Modulating Drugs, *ESAs* Erythropoiesis Stimulating Agents

**Table 2:** Predictors of anemia response to specific drugs in patients with primary myelofibrosis receiving these agents as first-line treatment

Baseline characteristics	HU (*n* = 38)	Response (*n*; %)	*P* value	JAK2 inhibitors (*n* = 18)	Response (*n*; %)	*P* value	IFN-α (*n* = 16)	Response (*n*; %)	*P* value	IMiDs (*n* = 50)	Response (*n*; %)	*P* value	Androgens (*n* = 18)	Response (*n*; %)	*P* value	ESAs (*n* = 54)	Response (*n*; %)	*P* value
*Age*																		
>70 years; *n (%)*	8 (21)	1 (12)	1	5 (28)	2 (40)	1	*2 (12)	0	1	15 (30)	7 (47)	1	6 (33)	1 (17)	0.6	21 (39)	12 (57)	1
≤70 years; *n (%)*	30 (79)	5 (17)		13 (72)	5 (38)		14 (88)	3 (21)		35 (70)	15 (43)		12 (67)	5 (42)		33 (61)	20 (61)	
*Sex*																		
Males; *n (%)*	21 (55)	3 (14)	1	11 (61)	3 (27)	0.3	7 (44)	0	0.2	35 (70)	14 (40)	0.5	14 (78)	4 (29)	0.5	33 (61)	19 (58)	0.7
Females; *n (%)*	17 (45)	3 (18)		7 (39)	4 (57)		9 (56)	3 (33)		15 (30)	8 (53)		4 (22)	2 (50)		21 (39)	13 (62)	
*Moderate/severe anemia (sex-adjusted)*																		
Presence; *n (%)*	21 (55)	4 (19)	0.6	9 (50)	4 (44)	1	9 (56)	0	0.06	44 (81)	20 (45)	0.6	16 (89)	6 (37)	0.5	39 (74)	20 (51)	0.1
Absence; *n (%)*	17 (45)	2 (12)		9 (50)	3 (33)		7 (44)	3 (43)		6 (19)	2 (33)		*2 (11)	0		14 (26)	11 (79)	
*Transfusion dependency*																		
Presence; *n (%)*	13 (34)	2 (15)	1	7 (39)	3 (43)	1	7 (44)	0	0.2	34 (68)	15 (44)	1	9 (50)	3 (33)	1	25 (46)	10 (40)	**0.01**
Absence; *n (%)*	25 (66)	4 (16)		11 (61)	4 (36)		9 (56)	3 (33)		16 (32)	7 (44)		9 (50)	3 (33)		29 (54)	22 (76)	
*Platelets*																		
<100 × 10^9^/L; *n (%)*	6 (16)	0	0.5	5 (28)	1 (20)	0.5	4 (25)	1 (25)	1	15 (30)	9 (60)	0.2	6 (33)	1 (17)	0.6	11 (20)	5 (45)	0.3
≥100 × 10^9^/L; *n (%)*	32 (84)	6 (19)		13 (72)	6 (46)		12 (75)	2 (17)		35 (70)	13 (37)		12 (67)	5 (42)		43 (80)	27 (63)	
*Leukocytes*																		
>25 × 10^9^/L; *n (%)*	8 (21)	1 (12)	1	3 (17)	0	0.2	3 (19)	2 (67)	0.07	*1 (2)	0	1	3 (17)	1 (33)	1	7 (13)	4 (57)	1
≤25 × 10^9^/L; *n (%)*	30 (79)	5 (17)		15 (83)	7 (47)		13 (81)	1 (8)		49 (98)	22 (45)		15 (83)	5 (33)		46 (87)	27 (59)	
*Circulating blasts*																		
≥2%; *n (%)*	8 (21)	0	0.3	6 (35)	1 (17)	0.3	7 (44)	0	0.2	13 (26)	3 (23)	0.1	6 (33)	3 (50)	0.3	15 (28)	8 (53)	0.7
<2%; *n (%)*	30 (79)	6 (20)		11 (65)	5 (45)		9 (56)	3 (33)		37 (74)	19 (51)		12 (67)	3 (25)		39 (72)	24 (62)	
*Constitutional symptoms*																		
Presence; *n (%)*	13 (34)	3 (23)	0.3	6 (33)	3 (50)	0.6	3 (19)	0	1	15 (30)	6 (40)	0.7	5 (28)	1 (20)	0.6	17 (31)	10 (59)	1
Absence; *n (%)*	25 (66)	3 (12)		12 (67)	4 (33)		13 (81)	3 (23)		35 (70)	16 (46)		13 (72)	5 (38)		37 (69)	22 (59)	
*Palpable splenomegaly*																		
Presence; *n (%)*	30 (79)	5 (17)	1	12 (67)	7 (58) 0	**0.03**	13 (81)	3 (23) 0	1	39 (78)	17 (44)	1	12 (71)	4 (33)	1	39 (72)	23 (59)	1
Absence; *n (%)*	8 (21)	1 (12)		6 (33)			3 (19)			11 (22)	5 (45)		5 (29)	1 (20)		15 (28)	9 (60)	
*Favorable karyotype*																		
Presence; *n (%)*	26 (68)	3 (12)	0.3	15 (83)	5 (33)	0.5	14 (88)	3 (21)	1	35 (70)	15 (43)	1	15 (83)	4 (27)	0.2	44 (81)	27 (61)	0.7
Absence; *n (%)*	12 (32)	3 (25)		3 (27)	2 (67)		*2 (12)	0		15 (30)	7 (47)		3 (17)	2 (67)		10 (19)	5 (50)	
*Unfavorable karyotype*																		
Presence; *n (%)*	7 (18)	2 (29)	0.3	3 (17)	2 (67)	0.5	*0	-	-	11 (22)	5 (45)	1	*2 (11)	1 (50)	1	7 (13)	4 (57)	1
Absence; *n (%)*	31 (82)	4 (13)		15 (83)	5 (33)		16 (100)	3 (19)		39 (78)	17 (44)		16 (89)	5 (31)		47 (87)	28 (60)	
*VHR karyotype*																		
Presence; *n (%)*	5 (13)	1 (20)	1	*0	-	-	*2 (12)	0	1	4 (8)	2 (50)	1	*1 (6)	1 (100)	0.3	3 (6)	1 (33)	0.5
Absence; *n (%)*	33 (87)	5 (15)		18 (100)	7 (39)		14 (88)	3 (21)		46 (92)	20 (43)		17 (94)	5 (29)		51 (94)	31 (61)	
*JAK2 mutationv*																		
Presence; *n (%)*	23 (61)	4 (17)	1	12 (67)	4 (33)	0.6	9 (56)	2 (22)	1	29 (58)	14 (48)	0.5	8 (44)	1 (12)	0.1	31 (57)	16 (52)	0.2
Absence; *n (%)*	15 (39)	2 (13)		6 (33)	3 (50)		7 (44)	1 (14)		21 (42)	8 (38)		10 (56)	5 (50)		23 (43)	16 (70)	
*CALR type-1/like mutation*																		
Presence; *n (%)*	8 (21)	2 (25)	0.5	3 (17)	1 (33)	1	5 (31)	1 (20)	1	13 (26)	4 (31)	0.2	*2 (11)	1 (50)	1	7 (13)	4 (57)	1
Absence; *n (%)*	30 (79)	4 (13)		15 (83)	6 (40)		11 (69)	2 (18)		37 (74)	18 (49)		16 (89)	5 (31)		47 (87)	28 (60)	
*CALR type-2/like mutation*																		
Presence; *n (%)*	*2 (5)	0	1	*	-	-	*1 (6)	0	1	*0	-	-	*1 (6)	1 (100)	0.3	3 (6)	2 (67)	1
Absence; *n (%)*	36 (95)	6 (17)		18 (100)	7 (39)		15 (94)	3 (20)		50 (100)	22 (44)		17 (94)	5 (29)		51 (94)	30 (59)	
*MPL mutation*																		
Presence; *n (%)*	*2 (5)	0	1	*1 (6)	0	1	*1 (6)	0	1	4 (8)	2 (50)	1	3 (17)	1 (33)	1	6 (11)	4 (67)	1
Absence; *n (%)*	36 (95)	6 (17)		17 (94)	7 (41)		15 (94)	3 (20)		46 (92)	20 (43)		15 (83)	5 (33)		48 (89)	28 (58)	
*Triple-negative*																		
Presence; *n (%)*	3 (8)	0	1	*2 (11)	2 (100)	0.1	*0	-	-	4 (8)	2 (50)	1	4 (22)	2 (50)	0.5	7 (13)	6 (86)	0.2
Absence; *n (%)*	35 (92)	6 (17)		16 (89)	5 (31)		16 (100)	3 (19)		46 (92)	20 (43)		14 (78)	4 (29)		47 (87)	26 (55)	
*ASXL1 mutation*																		
Presence; *n (%)*	17 (45)	1 (6)	0.9	9 (50)	2 (22)	0.3	8 (50)	1 (12)	1	21 (42)	8 (38)	0.5	4 (22)	2 (50)	0.5	23 (43)	14 (61)	1
Absence; *n (%)*	21 (55)	5 (24)		9 (50)	5 (56)		8 (50)	2 (25)		29 (58)	14 (48)		14 (78)	4 (29)		31 (57)	18 (58)	
*SRSF2 mutation*																		
Presence; *n (%)*	9 (24)	2 (22)	0.6	6 (33)	2 (33)	1	*2 (12)	0	1	8 (16)	4 (50)	0.7	*0	-	-	6 (11)	4 (67)	1
Absence; *n (%)*	29 (76)	4 (14)		12 (67)	5 (42)		14 (88)	3 (21)		42 (84)	18 (43)		18 (100)	6 (33)		48 (89)	28 (58)	
*EZH2 mutation*																		
Presence; *n (%)*	5 (13)	0	0.5	*0	-	-	*1 (6)	1 (100)	0.1	*1 (2)	0	1	*2 (11)	1 (50)	1	3 (6)	2 (67)	1
Absence; *n (%)*	33 (87)	6 (18)		18 (100)	7 (39)		15 (94)	2 (13)		49 (98)	22 (45)		16 (89)	5 (31)		51 (94)	30 (59)	
*IDH1 mutation*																		
Presence; *n (%)*	*2 (5)	1 (50)	0.2	*0	- 7 (39)	-	*0	-	-	*0	-	-	*0	-	-	*2 (4)	1 (50)	1
Absence; *n (%)*	36 (95)	5 (14)		18 (100)			16 (100)	3 (19)		50 (100)	22 (44)		18 (100)	6 (33)		52 (96)	31 (60)	
*IDH2 mutation*																		
Presence; *n (%)*	3 (8)	0	1	*1 (6)	1 (100)	0.3	*0	-	-	*2 (4)	1 (50)	1	*0	-	-	3 (6)	2 (67)	1
Absence; *n (%)*	35 (92)	6 (17)		17 (94)	6 (35)		16 (100)	3 (19)		48 (96)	21 (44)		18 (100)	6 (33)		51 (94)	30 (59)	
*U2AF1* Q157																		
Presence; *n (%)*	3 (8)	0	1	*1 (6)	0	1	*0	-	-	8 (16)	3 (37)	1	4 (22)	1 (25)	1	9 (17)	5 (56)	1
Absence; *n (%)*	35 (92)	6 (17)		17 (94)	7 (41)		16 (100)	3 (19)		42 (84)	19 (45)		14 (78)	5 (36)		45 (83)	27 (60)	
*High molecular risk mutations*																		
Presence; *n (%)*	24 (63)	3 (12)	0.6	12 (67)	4 (33)	0.6	10 (62)	2 (20)	1	31 (62)	11 (35)	0.1	7 (39)	3 (43)	0.6	31 (57)	19 (61)	0.7
Absence; *n (%)*	14 (37)	3 (21)		6 (33)	3 (50)		6 (38)	1 (17)		19 (38)	11 (58)		11 (61)	3 (27)		23 (43)	13 (57)	

The values in bold indicate a significant *P* value ( < 0.05)

The values preceded by asterisk indicate an insufficient number of patients in the sample ( ≤ 2)

*JAK2* Janus kinase 2 *CALR* Calreticulin *MPL* MPL proto-oncogene, *ASXL1* additional sex combs like 1, *SRSF2* Serine/arginine-rich splicing factor 2, *U2AF1* U2small nuclear RNA auxiliary factor 1, *EZH2* enhancer of zeste homolog 2, *IDH1/2* isocitrate dehydrogenase ½, *HU* Hydroxyurea, *IFN-α* Interferon α, *IMiDs* Immuno-Modulating Drugs, *ESAs* Erythropoiesis Stimulating Agents

Spleen response to HU was more likely in the presence of *ASXL1* (40% vs 17%; *p* = 0.06) or *SRSF2* (47% vs 22%; *p* = 0.1) mutations, while none of eight patients with either *U2AF1*Q157 or *IDH2* mutations responded, none of five patients with VHR karyotype, and only one (9%) of 11 patients with platelet count < 100 × 10^9^/l responded. Anemia responses to HU were infrequent. Overall response rate to HU was predicted by the absence of *U2AF1*Q157 mutations (64% vs 0%; *p* = 0.007).

Spleen response to JAK2 inhibitors was more likely in female patients (87% vs 50%; *p* = 0.01), absence of triple-negative mutational status (72% vs 0%; *p* = 0.01), and presence of *CALR* type 1/like mutations (90% vs 57%; *p* = 0.06). *ASXL1* (65% vs 65%; *p* = 1.0) or *SRSF2* (54% vs 70%; *p* = 0.4) mutations did not influence spleen response to JAK2 inhibitors. Anemia responses to JAK2 inhibitors were largely unpredictable. In order to further verify the aforementioned-observed associations, we accessed data from a previous formal clinical trial of momelotinib (JAK2 inhibitor). Data from this trial showed that among 91 evaluable patients, spleen response was higher in the presence of *CALR* mutations (73% vs 37%; *p* = 0.009) and female sex (49% vs 39%, *p* = NS).

Although significant differences were not apparent, spleen response to IFN-α was unlikely in the presence of ≥ 2% circulating blasts (none of six patients responded) or presence of *ASXL1* mutations (none of seven patients responded). IFN-α was often ineffective for the treatment of anemia. Spleen responses to treatment with IMiDs, androgens, or ESAs were unusual, while anemia response to all three agents was not predicted by either genetic or clinical markers.

Our observations, which require additional examination in a prospective setting, show a limited value of genetic and clinical markers in predicting response to currently available drugs for PMF (consistent with the non-specific mechanism of action for these drugs). The study also confirmed the possibility that *CALR* mutations and female sex predict favorable spleen response to JAK2 inhibitors and suggest *U2AF1*Q157 mutations predict unfavorable spleen response to HU, as previously proposed in formal clinical trials.

## Electronic supplementary material

Supplementary table
