# Supplementary material for: Genetic predictors of response to specific drugs in primary myelofibrosis
Source: Blood Cancer J. 2018 Nov 19;8(12):120. doi: 10.1038/s41408-018-0158-4 (PMC6242902; doi:10.1038/s41408-018-0158-4)
Supplement: Supplementary file 1 — Supplementary table [file 41408_2018_158_MOESM1_ESM.docx]

**Supplementary table: Baseline characteristics of 432 cytogenetically- and molecularly-annotated patients with primary myelofibrosis evaluated for specific drug treatment response**

| Baseline Characteristics | All Patients  (*n*=432) | Received Treatment  (*n*=333) | No Treatment or Incomplete treatment response data  (*n*=99) |
| --- | --- | --- | --- |
| Age in years; median (range) | 63 (22-88) | 64 (22-84) | 59 (26-88) |
| Age >70 years; *n (%)* | 103 (24) | 82 (25) | 21 (21) |
| Sex (male); *n (%)* | 285 (66) | 226 (68) | 59 (60) |
| Moderate/severe anemia (sex adjusted); *n (%)*  “N” evaluable= 431 (99%) | 222 (52) | 192 (58) | 30 (30) |
| Transfusion dependency; *n (%)* | 132 (31) | 118 (35) | 14 (14) |
| Platelets <100 x10^9^/L; *n (%)* | 88 (20) | 65 (20) | 23 (23) |
| Leukocytes >25x10^9^/L; *n (%)*  “N” evaluable= 431 (99%) | 59 (14) | 47 (14) | 12 (12) |
| Circulating blasts >1%; *n (%)*  “N” evaluable=430 (99%) | 123 (29) | 98 (30) | 25 (25) |
| Presence of constitutional symptoms; *n (%)* | 118 (27) | 102 (31) | 16 (16) |
| Presence of palpable splenomegaly; *n (%)*  “N” evaluable=428 (99%) | 320 (75) | 253 (77) | 67 (68) |
| Karyotype risk category |  |  |  |
| Favorable; *n (%)* | 334 (77) | 251 (76) | 83 (84) |
| Unfavorable; *n (%)* | 75 (18) | 61 (18) | 14 (14) |
| Very high risk; *n (%)* | 23 (5) | 21 (6) | 2 (2) |
| Absence of favorable karyotype; *n (%)* | 98 (23) | 82 (24) | 16 (16) |
| Driver mutations |  |  |  |
| *JAK2; n (%)* | 243 (56) | 197 (59) | 46 (47) |
| *CALR type-1/like; n (%)* | 93 (22) | 66 (20) | 27 (27) |
| *CALR type-2/like; n (%)* | 19 (4) | 13 (4) | 6 (6) |
| *MPL; n (%)* | 32 (7) | 23 (7) | 9 (9) |
| Triple-negative; *n (%)* | 45 (11) | 34 (10) | 11 (11) |
| Absebnce of CALR type-1/like; *n (%)* | 339 (78) | 267 (80) | 72 (73) |
| High risk mutations |  |  |  |
| *ASXL1, n (%)* | 174 (40) | 151 (45) | 23 (23) |
| *SRSF2, n (%)* | 63 (15) | 59 (18) | 4 (4) |
| *EZH2, n (%)* | 17 (4) | 14 (4) | 3 (3) |
| *IDH1, n (%)* | 8 (1) | 7 (2) | 1 (1) |
| *IDH2, n (%)* | 17 (4) | 13 (4) | 4 (4) |
| *U2AF1* Q157*, n (%)* | 42 (10) | 34 (10) | 8 (8) |
| Presence of high molecular risk, n (%) | 233 (54) | 201 (60) | 32 (32) |
| GIPSS prognostic score risk category |  |  |  |
| Low; *n (%)* | 41 (9) | 22 (7) | 19 (19) |
| Intermediate-1; *n (%)* | 165 (38) | 116 (35) | 49 (50) |
| Intermediate-2; *n (%)* | 128 (30) | 108 (32) | 20 (20) |
| High; *n (%)* | 98 (23) | 87 (26) | 11 (11) |
| MIPSS70-plus version 2 prognostic score risk category |  |  |  |
| Very low; *n (%)* | 22 (5) | 7 (2) | 15 (15) |
| Low; *n (%)* | 77 (18) | 45 (14) | 32 (33) |
| Intermediate; *n (%)* | 91 (21) | 71 (21) | 20 (20) |
| High; *n (%)* | 178 (41) | 154 (46) | 24 (24) |
| Very high; *n (%)* | 64 (15) | 56 (17) | 8 (8) |

Abbreviations: *JAK2*, Janus kinase 2; *CALR*, Calreticulin; *MPL*,MPL proto-oncogene; *ASXL1*, additional sex combs like 1; *SRSF2* Serine/arginine-rich splicing factor 2; *U2AF1*, U2small nuclear RNA auxiliary factor 1; *EZH2*, enhancer of zeste homolog 2; *IDH1/2*, isocitrate dehydrogenase ½; GIPSS: genetically inspired prognostic scoring system; MIPSS70 plus, karyotype-enhanced mutation international prognostic score system for transplant-age.

Revised cytogenetic risk stratification: “very high risk (VHR)”—single/multiple abnormalities of −7, i(17q), inv(3)/3q21, 12p−/12p11.2, 11q−/11q23, +21, or other autosomal trisomies, not including +8/+9; “favorable”—normal karyotype or sole abnormalities of 13q−, +9, 20q−, chromosome 1 translocation/duplication or sex chromosome abnormality including—Y; “unfavorable”—all other abnormalities

MIPSS70-plus, karyotype-enhanced mutation international prognostic score system for transplant-age: this score considers six clinical risk variables (hemoglobin < 10 g/dL;, leukocytes > 25 × 109/L; platelets < 100 × 109/L; circulating blasts ≥ 2%; marrow fibrosis grade ≥ 2; and constitutional symptoms), five high molecular risk mutations (ASXL1, SRSF2, EZH2, IDH1, and IDH2), one favorable mutation (CALR type 1/like) and a two-tiered cytogenetic risk variable (unfavorable v favorable)

GIPPS, Genetically Inspired Prognostic Scoring System: this score considers only the prognostic relevance of VHR karyotype, unfavorable karyotype and certain mutations including the prognostically favorable type 1/like CALR mutation and the prognostically unfavorable ASXL1, SRSF2, and U2AF1Q157 mutations.
